# A mixture of pomegranate peels and propolis extracts reduces inflammation associated with rheumatoid arthritis *in vivo*

**DOI:** 10.3389/fphar.2026.1910652

**Published:** 2026-10-07

**Authors:** Maria Laura Bellone, Angela Carmelita Abate, Paola Di Pietro, Valentina Parisi, Michele D’Angiolo, Federica Prosperi, Claudio Pisano, Albino Carrizzo, Alessandra Braca, Fabrizio Dal Piaz, Marinella De Leo, Nunziatina De Tommasi

**Affiliations:** 1 Department of Pharmacy, University of Salerno, Fisciano, Italy; 2 Department of Medicine, Surgery and Dentistry, University of Salerno, Baronissi, Italy; 3 Department of Pharmacy, University of Pisa, Pisa, Italy; 4 Animal Facility Unit, Biogem, Ariano Irpino, Avellino, Italy; 5 Vascular Physiopathology Unit, IRCCS Neuromed, Pozzilli, Italy; 6 Center for Instrument Sharing University of Pisa (CISUP), University of Pisa, Pisa, Italy

**Keywords:** cytokines, immunohistochemical analysis, *in vivo* assays, inflammation, M1 polarization, pomegranate, propolis, rheumatoid arthritis

## Abstract

**Background:**

Rheumatoid arthritis (RA) is a chronic condition characterized by the infiltration of immune and inflammatory cells into the synovial membranes, which often leads to severe damage to cartilage and bone. In spite of their efficacy, drug-based therapies produce severe side effects. Therefore, there remains an unmet need for non-pharmacological treatments with a low toxicity profile and limited costs. Propolis has been used since ancient times for the treatment of wounds, ulcers, infections, eczemas, myalgia, and rheumatism. Similarly, the traditional use of all parts of pomegranate (*Punica granatum* L.) is documented across Egyptian, Persian, and Chinese cultures for the treatment of infections and inflammations.

**Purpose:**

This study aimed at the development of preparations containing bioactive compounds derived from propolis and pomegranate peels, inspired by traditional recipes used in rural areas of southern Italy, to validate their efficacy in counteracting the inflammation associated with RA.

**Materials and methods:**

A preliminary screening led to the selection of the most promising preparation, composed by propolis and pomegranate peel extracts in a ratio 1:2 (Pp1:2). It underwent a complete chemical characterization by both qualitative and quantitative LC-MS analyses and was tested on a collagen-induced arthritis (CIA) mouse model.

**Results:**

*In vitro* tests showed that Pp1:2 treatment of M1 stimulated by LPS/IFNγ reduces cell polarization; furthermore, it reverts the pro-inflammatory effects induced by IL-1β on synoviocytes. *In vivo* experiments, conducted by administering Pp1:2 orally to a suitable murine model, confirmed the ability of the preparation to reduce sensibly RA-like inflammatory effects, as inferred by the change observed in mice paws volume and other RA-related parameters. Furthermore, histological analyses revealed a reduction in the number of M1 macrophages in mice paws following Pp1:2 treatment, and an attenuation of the pathological remodeling of collagen IV and of ICAM-1 expression, compared to untreated controls.

## 1 Introduction

Rheumatoid arthritis (RA) is a chronic systemic autoimmune disease (Smolen et al., 2016). Pathologically, RA presents a dense infiltration of immune and inflammatory cells into the synovial membranes of the joints, leading to progressive cartilage and bones erosion (Chauhan et al., 2023). The pathogenesis of RA is multifactorial and highly complex, involving the dysregulated infiltration of leukocytes, the imbalance of macrophage polarization—specifically the skewing toward pro-inflammatory M1 macrophages over anti-inflammatory M2 phenotypes—the sustained release of pro-inflammatory cytokines, and the abnormal proliferation of synovial tissue (Zheng et al., 2024). Clinically, symptoms vary depending on the stage of the disease and patients with advanced RA or severe untreated RA may even experience physical disability. The extensive knowledge of the physiopathology of the RA has convinced the scientific community to pursue the multi-targeted approach thus to hit the main molecular players with specific therapeutic agents ensuring the lowest off-target. Corticosteroids, non-steroidal anti-inflammatory drugs (NSAIDs), disease-modifying antirheumatic drugs (DMARDs), and additional biological agents represent the main conventional treatments of RA (Smolen et al., 2023; Bao et al., 2024). Nevertheless, these drugs only serve to limit the progression of RA rather than induce a remission of the pathology and many adverse side effects are related to their use (Liang et al., 2024). One of the solutions to address these critical aspects consists in the development of new preparations with potential therapeutic application adapted to the long-term therapy of chronic diseases. For this purpose, the combination of natural products and conventional therapies appears to be one of the most promising solutions.

Based on ethnopharmacological knowledge, propolis, a resinous material produced by the honeybees (*Apis mellifera* L.) from exudates and secretions of different plant species, has been used for centuries for its medicinal properties. The ancient Egyptians, Persians, Greeks, and Romans used propolis extensively as a remedy for external and internal wounds, ulcers, abscesses, eczema, myalgia, rheumatism, and infections. In the Mediterranean regions, including southern Italy, propolis has long been incorporated into traditional folk medicine, where it has been used as a natural remedy for respiratory tract infections, sore throat, oral and skin inflammations, and wound healing (Castaldo and Capasso, 2002; Kuropatnicki et al., 2013). As other remarkable example of very ancient remedy, pomegranate (*Punica granatum* L., Lythraceae) has been widely used as a medicine for thousands of years across different cultures, such as ancient Egyptians and Persians as well as in Traditional Chinese Medicine and Ayurvedic Medicine. Historical records indicate that various parts of the plant, including the fruit, peel, and leaves were traditionally employed for the treatment of gastrointestinal disorders, parasitic infections, inflammation, wounds, and other ailments. In the Mediterranean basin, where pomegranate cultivation has a long-standing tradition, the plant has been extensively used in folk medicine and is deeply rooted in local ethnobotanical practices. In southern Italy, particularly in the rural areas, pomegranate preparations have traditionally been used for their astringent and antiseptic activities. All parts of pomegranate, including leaves, bark, flowers and all parts of fruits (peels, arils, and seeds) have been reported as traditional remedies against several diseases such as gastrointestinal disorders, pain, cough, ulcers, wounds, and cancer (Lansky and Newman, 2007; Ge et al., 2021).

Propolis and pomegranate have been widely investigated for their possible use in inflammation-related pathologies (Cordiano et al., 2024; Zulhendri et al., 2022). Their main metabolites, including polyphenols, flavonoids, alkaloids, terpenes and other compounds, are gaining more attention given their capability to reduce inflammatory status. Propolis exerts anti-inflammatory and immunomodulatory effects through the action of flavonoids and caffeic acid phenetyl ester (CAPE), which has been identified as a potent inhibitor of NF-κB (Ansorge et al., 2003; Smolen et al., 2016). Pomegranate is a source of polyphenolic compounds, such as punicalagin and punicalin, able to improve the inflammatory status by regulating several pathways, such as NF-κB, MAPK and PI3K/Akt/mTOR signaling pathways (Rasheed et al., 2009; Xu et al., 2021). The presence of these bioactive metabolites in the common fruits, both edible part and peel by-products, makes them value-added products in developing nutraceutical products and functional foods. Besides, the revalorization of industrial waste improves circular economy processes through the aim of obtaining a food supplement endowed with a high translational value. In our previous work, a botanical drug consisting of propolis, pomegranate peel, and grape pomace extracts alleviated the severity of collagen-induced arthritis (CIA) clinical symptoms, probably due to the high polyphenols content of the preparation (Parisi et al., 2020). Based on these findings, this study focused on analyzing the potential of pomegranate peels—commonly regarded as industrial waste—as a valuable source of anti-inflammatory compounds to be used in combination with propolis. Therefore, different preparations composed by propolis combined with pomegranate juice or pomegranate peels-extracts were preliminary tested on macrophages to evaluate their actual anti-inflammatory efficacy. Results obtained using a mixture of propolis and pomegranate peels extract in a ratio 1:2 (Pp1:2) were very promising and comparable to those provided by preparations containing pomegranate juice. Therefore, Pp1:2 was tested on a CIA mouse model showing to be able to reduce RA-related inflammation and symptoms of the pathology. The present work reported a comprehensive account of the chemical composition of the mixture, confirmed the actual bioavailability of bioactive compounds present in the mixture, and described at a molecular level the effects that the treatment produces in both *in vivo* and *in vitro* RA models.

## 2 Materials and methods

### 2.1 Chemicals

Analytical grade solvents were purchased from VWR (Milano, Italy). Romil UHPLC-grade MeOH, H_2_O, and HCOOH were acquired from DelTek (Pozzuoli, Italy). Pure reference standards (purity >98.0%), pinobanksin, pinocembrin, caffeic acid phenethyl ester (CAPE), galangin, chrysin, punicalagin, and ellagic acid were purchased from DBA Italia s. r.l. (Milano, Italy).

### 2.2 Materials

Propolis (1 kg) was provided during 2021 by the Consortium of Sannio-Irpinia Apicoltura (Capriglia Irpina, Avellino, Italy). Pomegranate fresh fruits (12 kg) were supplied during 2021 by the Irpinian organic farm “Giovomel” (Aiello del Sabato, Avellino, Italy). The pomegranate juice was achieved by manual squeezer obtaining 370 mL/kg of fresh fruits that was freeze-dried to be used for preparations. Pomegranate peels were separated from fruits and dried in a conventional oven at 40 °C, then powdered by using a MF 10 basic Microfine grinder drive (IKA-Werke, Staufen, Germany), obtaining 2.0 kg of dried material. This material was used for the whole study. THP-1 (human acute monocytic leukaemia) and SW982 (human synovial sarcoma) cells were obtained from American Type Culture Collection (ATCC). CCK-8, Cell Counting Kit-8 was purchased from Dojindo Molecular Technologies (Kumamoto, Japan). Interleukin 1-β was purchased from Proteintech Group, Inc. (HQ in North America). For the measurement of cellular viability and ELISA assay, the spectrophotometer Multiskan GO was used (Thermo Scientific, Waltham, MA, United States). The analysis of interleukin levels in cell supernatants was performed using ELISA KIT Diaclone Research (Besancon, France).

### 2.3 Extraction and design of preparations based on combined extracts

Propolis and powdered pomegranate dried peels (10 g each) were separately extracted with 100 mL of EtOH-H_2_O 70–30 (v/v) by Ultrasound Assisted Extraction–Bath (LBS2 (Falc Instruments s. r.l., Treviglio, Italy) for 15 min at 59 Hz and 40 °C. After removal of the solvent using a Rotavapor® (Buchi Italia S. r.l., Cornaredo, Italy), 4.7 g and 2.8 g of dried extracts were obtained from propolis and pomegranate peel, respectively. Four different preparations were prepared mixing propolis extract (P) and pomegranate dried juice (j) or peel dried extract (p) in different proportions (Pj1:1, Pp1:2, Pp1:4, and Pp2:1) and were used for chemical analyses and biological assays. All the preparations were stored at room temperature in amber vials until use.

### 2.4 High resolution LC-ESI-MS analyses

The chemical profile of Pp1:2 preparation was investigated by a Vanquish Flex UHPLC System coupled to a diode array detector (DAD) and a high-resolution mass spectrometry (HR-MS) Q Exactive Plus Orbitrap-based FT-MS equipped with an electrospray ionization (ESI) source (Thermo Fisher Scientific Inc., Bremen, Germany). Pp1:2 preparation was dissolved in MeOH (2 mg/mL concentration) centrifuged for 5 min at 2710 × *g*, and 5 µL of supernatant were injected into the LC-MS system. LC/MS/MS analysis was performed as reported elsewhere (Vitiello et al., 2024). The most representative constituents of Pp1:2 preparation were quantified by using pure external standards solutions in a concentration range of 0.03–0.01 mg/mL, successively injected in triplicate into the LC-MS for constructing calibration curves.

### 2.5 Cell culture

Human monocytic cell line (THP-1) and human synovial sarcoma cell line (SW982) cells were grown respectively in RPMI-1640 and DMEM, both supplemented with 1% penicillin/streptomycin, 1% L-glutamine, 1% sodium pyruvate and 10% fetal bovine serum (FBS). Cells were maintained under a humidified atmosphere with 5% CO_2_ at 37 °C. Macrophages M0 were obtained by treating THP-1 with 100 nM of phorbol-12-myristate-13-acetate (PMA) for 24 h. Cell differentiation was enhanced by replacing the PMA-containing media with fresh complete media for 24 h.

### 2.6 Cellular viability assay

SW982 cells were seeded in 96-well plates (5 × 10^3^ cells/well) and, the day after, were treated with 12.5–25-50 μg/mL Pp1:2, with or without IL-1β (0.1 μg/mL), and incubated for 24 h. Absorbance was measured at 550 nm using Multiskan GO. Experiments were performed twice and analyzed in three technical replicates. Each measured value was reported as optical density (OD) normalized to the OD average for vehicle samples.

### 2.7 Preliminary screening in macrophages M0 by Enzyme-Linked Immunosorbent Assay (ELISA)

THP-1 were seeded in 6-well plates (1 × 10^6^ cells/well), differentiated in macrophages M0, as described above and treated with the mixture Pp1:2 (12.5–25-50 μg/mL) and LPS (0.1 μg/mL) or vehicle for 24 h. The conditioned media were harvested and ELISA assay was carried out according to the manufacturer’s instructions. Each measured value was reported as OD normalized to the OD average for IL1β-treated samples.

### 2.8 Anti-inflammatory evaluation of Pp1:2 mixture by Enzyme-Linked Immunosorbent Assay (ELISA)

For the anti-inflammatory evaluation in synovial cells, SW982 cells were seeded in 24-well plates (7 × 10^4^ cells/well). The day after, the cells were subjected to co-treatment with the mixture Pp1:2 (12.5–25-50 μg/mL) and IL-1β (5 ng/mL) for 3 h. The conditioned media were harvested and ELISA assay was carried out according to the manufacturer’s instructions. The samples were analyzed in technical duplicate. Mean OD value was calculated and reported as OD normalized to the OD average for IL-1β sample. The experiment was performed in biological duplicate.

### 2.9 *In vivo* study in collagen-induced arthritis (CIA) mouse model

The *in vivo* tests were performed at the Test Facility BIOGEM (Ministerial Authorization n° 47/2022-PR). Females 8–12 weeks old DBA1/J mice were purchased from Charles River France Les Oncins (Saint-Germain-Nuelles, France). The number of animals per group was selected based on previous experience with the collagen-induced arthritis (CIA) model and on previously published study (Parisi et al., 2020), which employed a comparable experimental design and group size (Supplementary Table S1). In addition, the selected sample size was considered consistent with the principle of Reduction within the 3Rs framework, aiming to minimize animal use while allowing evaluation of the biological effects of Pp1:2. The animal welfare was checked by trained personnel for any clinical sign of disease or other clinical or behavioral abnormalities. The animals were managed according to the Directive 2010/63/UE regarding the protection of animals used for experimental or other scientific purposes, enforced by the Italian decree n° 26 of 4 March 2014. Arthritis was induced by using collagen from chicken sternal cartilage Type II (Miller) (CII). Collagen was dissolved overnight in 10 mM acetic acid at a concentration of 2 mg/mL and then collagen and the adjuvant were injected in a 1:1 ratio (50 μL/mouse) at the base of the tail. Feed and water were provided *ad libitum* to all animals. Body weight was recorded for each animal weekly, starting from day 0 until the end of the study. The evaluation of the inflammation state was performed using the VEVO 2100 (VisualSonics) Power Doppler ultrasound system. In particular, changes in paw volume were evaluated weekly by using a plethysmometer. Edema was expressed as an increase in paw volume. Results were measured as change in paw volume (mL). Physical observations, including visual check on general appearance of the mice for health status were performed daily. In particular, starting from the development of the disease, each paw was evaluated and scored individually on a scale 0–4, with 4 indicating the most severe inflammation (Table 1).

**TABLE 1:** Scoring system for subjective evaluation of arthritis severity.

Severity score	Degree of inflammation
0	No evidence of erythema and swelling
1	Erythema and mild swelling confined to the tarsals or ankle joint
2	Erythema and mild swelling extending from the ankle to the tarsals
3	Erythema and moderate swelling extending from the ankle to metatarsal joints
4	Erythema and severe swelling encompass the ankle, foot and digits, or ankylosis of the limb

### 2.10 Treatment of CIA-induced mice with Pp1:2

Forty mice with CIA-induced inflammation were divided into 8 groups (GP1–GP8) and treated with different doses of Pp1:2 (100 mg/kg, 150 mg/kg, or 200 mg/kg), either at the time of inflammation induction or 23 days after induction, as shown in Supplementary Figure S1. Following collagen immunization, mice were randomly assigned to the eight experimental groups before the onset of clinical arthritis, as the study was designed to evaluate both the preventive and therapeutic effects of Pp1:2. Randomization was performed to ensure an unbiased allocation of animals across treatment groups. All animals were housed under identical environmental conditions, received standardized treatments, and were evaluated using predefined clinical endpoints to minimize potential experimental bias.

Pp1:2 mixture was administered by oral gavage (OS), considering a dose of 10 mL/kg. At the end of the study period, all animals were subjected to blood sampling under isoflurane anesthesia, from the submandibular vein, before sacrifice. Blood was collected in heparinized test tubes and then centrifuged at about 1500 × *g* for 10 min. Resulting plasma samples were stored at −80 °C. The animals were sacrificed by CO_2_ and subjected to paws sampling. In particular, one fore and one hind paw (from the same side of the animal) of each mouse were collected and fixed in 10% buffered formalin for immunohistochemistry analysis.

### 2.11 Histology

Hind paws were fixed in 10% buffered formalin and then decalcified for 3 weeks in 15% EDTA. Sagittal serial cryosections (6 μm) were prepared and stained with Safranin-O staining to assess cartilage damage, as previously described (Douni et al., 2003). Imaging analyses were performed using a Nikon Eclipse Ti-E inverted microscope (Melville, NY) equipped with a color digital camera (DS-Fi3, Nikon).

### 2.12 Immunofluorescence

Following treatment, SW982 and THP1-derived M0 cells seeded on 12 mm glass coverslips were fixed with 4% paraformaldehyde in 0.1 M PBS for 15 min at room temperature. For permeabilization, cells were incubated in PBS containing 0.005% saponin for 30 min. Both cell cultures and tissue cryosections were incubated overnight at 4 °C with specific primary IgG antibodies. Detection was performed using the following secondary antibodies for 1 h: Alexa Fluor 488–conjugated anti-rabbit IgG (Invitrogen, Cat# A11008) or DyLight 549–conjugated anti-rabbit IgG (Vector Laboratories, Cat# DI-1549). All samples were visualized using an inverted Leica TCS SP5 laser-scanning confocal microscope (Leica Microsystems). Fluorochromes were excited using the 488 nm and 543 nm laser lines. Image analysis was conducted using ImageJ software (NIH). For tissue evaluation, the entire paw section was scanned to identify and localize regions of positive immunoreactivity.

### 2.13 Metabolite analyses in mouse serum samples

The serum mouse samples were analysed by UHPLC-HR-Orbitrap/ESI-MS/MS to investigate the presence of specialized metabolites after administration of Pp1:2 preparation. Serum samples (50 µL) were mixed with 100 µL of acetonitrile, to precipitate proteins included in the plasma, then centrifugated for 5 min at 2710 × *g*. The supernatants were collected, dried in a vacuum concentrator Eppendorf Concentrator plus™ (Eppendorf srl, Milano, Italy) and successively added to 50 μL of plasma to be injected (5 µL) into the UHPLC-MS system. The solvent gradient was: 0–22 min, 5–55% B; 22–27 min 55% B isocratic mode; 27–35 min, 55%–80% B. Mass spectra were recorded in a scan range of 120–1500 *m/z* in both positive and negative ESI modes, using the same ionization parameters as previously described. Pinobanksin, chrysin, galangin, pinocembrin, ellagic acid, and CAPE were used as standards. For each molecule, solutions in a concentration range from 0.001 to 500 ng/mL were prepared and analysed. The resulting curves demonstrated good linearity, showing *R* ^2^ of 0.9992 for CAPE, 0.9998 for pinobanksin, 0.9949 for pinocembrin, 1.000 for chrysin and galangin, and 0.9998 for ellagic acid. Samples analyses were carried out in triplicate. Results were expressed as average measured concentration (ng/mL) ± standard deviation (SD).

### 2.14 Statistical analysis

Regarding cellular experiments, MTT and ELISA results are reported as the mean values ±SEM from two biological replicates experiments, performed in technical triplicate and duplicate, respectively. One-way ANOVA with Dunnett’s multiple comparisons test was used for statistical analysis of IL-6 assay of the different preparations on macrophages. For *in vivo* experiments, data are reported as the mean values ±SEM. One-way ANOVA with Tukey or Bonferroni *post hoc* comparison was used for comparisons between groups. One-way ANOVA followed by a *post hoc* test for linear trend was used to evaluate dose-dependent differences across groups. Statistical analysis was performed with GraphPad Prism software (version 8.0, GraphPad Software, San Diego, CA). Differences were considered significant when p < 0.05.

## 3 Results

### 3.1 The preparation Pp1:2 reduces inflammation in LPS-stimulated macrophages M0

Preliminarily, the potential anti-inflammatory effects of different preparations obtained by mixing propolis and pomegranate juice or peel dried extracts in different proportions (Pj1:1, Pp1:2, Pp1:4, and Pp2:1) were evaluated in THP1-derived macrophages M0. As IL-6 is a pro-inflammatory cytokine with a pivotal role in the pathophysiology of rheumatoid arthritis, the ability of the different extracts to reduce its secretion levels after M0 24 h treatment with LPS was monitored. Although all the four preparations induced an evident and dose-dependent reduction of IL-6 secretion confirming their anti-inflammatory properties (Figure 1), Pj1:1 and Pp1:2 showed almost equally promising effects. In order to develop an efficient yet low-cost formulation, one of the goals of our work was to explore the use of pomegranate peels—a byproduct of the agri-food industry—as an alternative source of bioactive compounds to juice. Therefore, based on the obtained results we decided to continue the study with Pp1:2.

**FIGURE 1:**
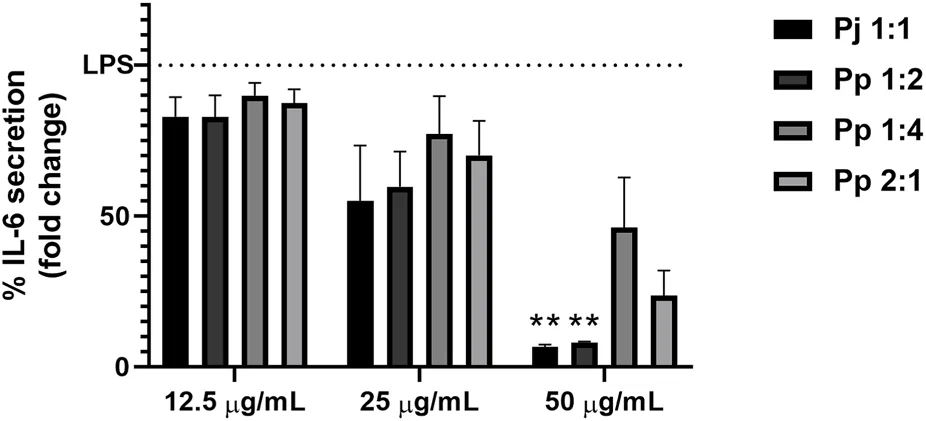
Effect of the different preparations on macrophages. Concentration of secreted IL-6 from THP1-derived macrophage cells subjected to a 24 h exposure LPS alone or in the presence of different concentrations of the preparations, measured by ELISA. The concentration of IL-6 secreted following the cells treatment with LPS alone was arbitrary set at 100 as dotted line. One-way ANOVA with Dunnett’s multiple comparisons test was used for statistical analysis of IL-6 assay of the different preparations on macrophages. **p < 0.01.

### 3.2 High resolution LC-MS/MS analyses of Pp1:2 confirmed the presence of the most active compounds from both the sources

The chemical composition of propolis is strongly dependent on the flora of the region where it is harvested. Furthermore, the extraction methods used on pomegranate peels determine the quality and quantity of the metabolites found in the extracts. Therefore, it was first necessary to conduct a comprehensive chemical characterization of Pp1:2, which was conducted by UHPLC-HR-Orbitrap/ESI-MS analysis, in both negative and positive ionization modes (Figure 2). Fifty-four molecules (Table 2) were tentatively identified, based on their HR MS and MS/MS spectra, chromatographic data, and previously reported information (Parisi et al., 2020). The identity of some compounds was confirmed through the injection of reference standards. Intriguingly, all the pomegranate metabolites (**1**–**9**, **11**, and **13**–**17**) were observed in the first part of the chromatogram (retention times <18 min) and only in negative ionization mode, while the propolis constituents (**18**–**54**) were more apolar and were detected in both ion modes, except for caffeic acid (**10**) and *p*-coumaric acid (**12**).

**FIGURE 2:**
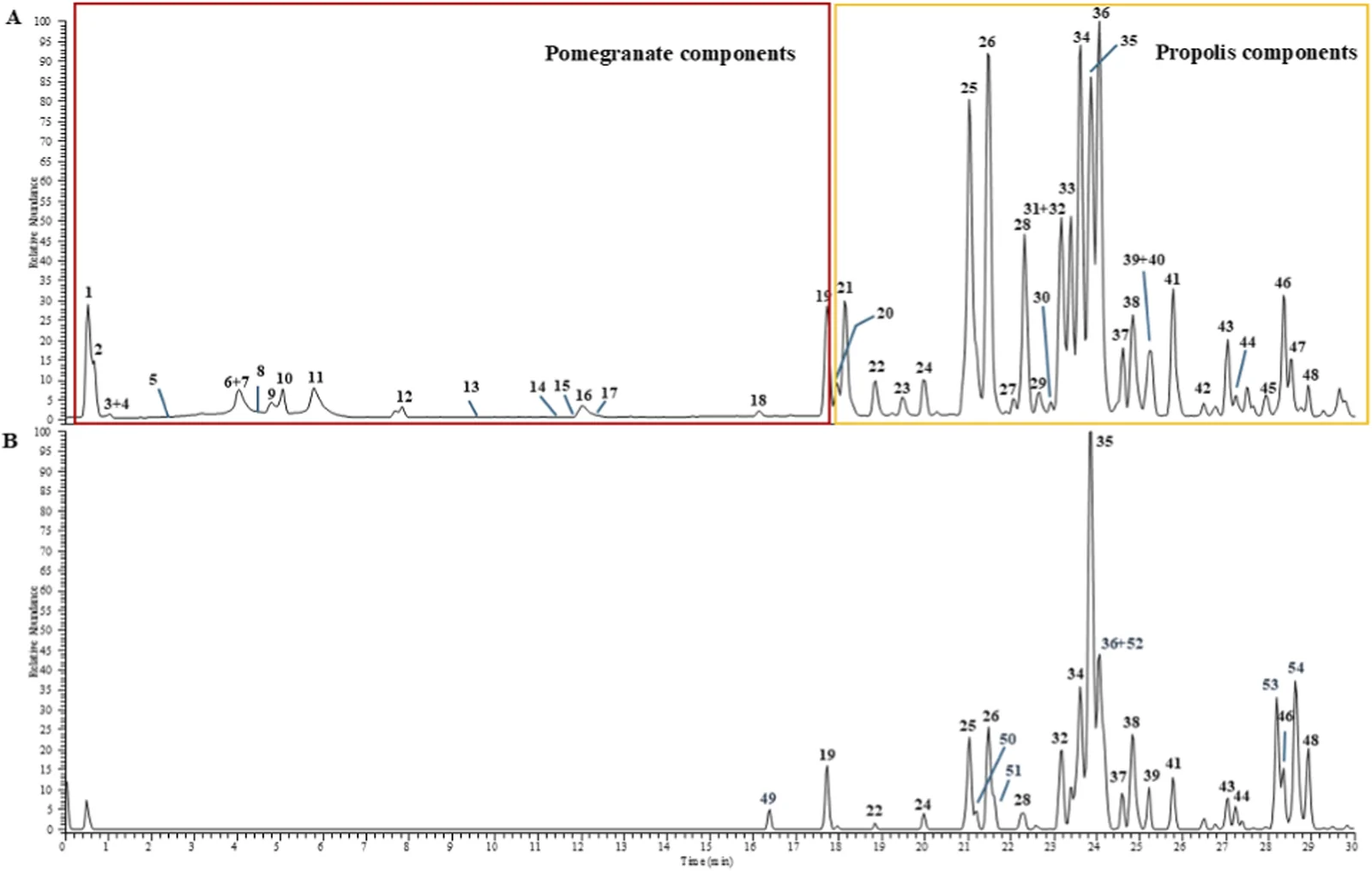
High-resolution LC/MS/MS analyses. Chemical profile of Pp1:2 preparation obtained by UHPLC-HR-Orbitrap/ESI-MS analyses registered in both negative **(A)** and positive **(B)** ion mode. Compound numbers correspond to those reported in Table 2.

**TABLE 2:** Chromatographic (*t*_R_ = retention time), full ESI-MS and MS/MS data of constituents identified in propolis-pomegranate peel (Pp1:2) preparation. The amount (g/100 g dry preparation) of the major metabolites is also reported.

Peak^a^	Compound	*t*_R_ (min)	[M – H]^-^(*m*/*z*)	ESI-MS/MS Product ions^b^	Molecular formula	Error (ppm)	Amount (g/100 g dry extract)^d^
Pomegranate peel metabolites							
1	Citric acid	0.7	191.0193	173.00, **111.01**, 87.00	C_6_H_8_O_7_	−2.2	-
2	HHDP-hexoside	0.8	481.0625	301.00, 275.02	C_20_H_18_O_14_	−0.2	1.6 ± 0.1
3	Gallic acid	1.0	169.0137	**125.02**, 97.03, 79.02, 69.03	C_7_H_6_O_5_	3.3	1.4 ± 0.1
4, 5	Galloyl-HHDP-gluconate I and II (lagerstannin C)	1.2	649.0685	497.06, **301.00**	C_27_H_22_O_19_	−0.4	0.15 ± 0.09
6	Punicalagin isomer I^e^ (HHDP-gallagyl-glucose)	4.0	541.0263^c^ ([M - 2H]^2‒^)	781.05, 600.99, 575.01, 301.00, 275.02	C_48_H_28_O_30_	−0.6	0.82 ± 0.06
7	Bis-HHDP-hexoside (pedunculagin I)	4.0	783.0694	481.06, **301.00**, 275.02	C_34_H_24_O_22_	−1	0.43 ± 0.05
8	Valoneic acid bilactone	4.3	469.0045	425.01, 407.00, **301.00,** 300.00, 299.00	C_21_H_10_O_13_	+0.8	0.41 ± 0.02
9	Bis-HHDP-hexoside (pedunculagin isomer II)	4.9	783.0694	481.06, **301.00**, 275.02	C_34_H_24_O_22_	−0.2	0.24 ± 0.09
11	Punicalagin isomer II^e^	5.8	541.0263^c^ ([M - 2H]^2‒^)	781.05, 600.99, 575.01, 301.00, 275.02	C_48_H_28_O_30_	−0.3	1.0 ± 0.3
13	Ellagic acid hexoside	9.4	463.0519	**301.00**	C_20_H_16_O_13_	−0.8	0.95 ± 0.09
14	Galloyl-HHDP-DHHDP-hexoside (granatin B)	11.5	951.0748	933.06, 765.06, 613.05, 445.04, **301.00**	C_41_H_28_O_27_	+1.0	0.36 ± 0.09
15	Ellagic acid pentoside	11.9	433.0416	301.00, 300.00	C_19_H_14_O_12_	+0.2	0.62 ± 0.03
16	Ellagic acid^e^	12.0	300.9987	257.01, 229.01, 185.02	C_14_H_6_O_8_	−2.3	4.8 ± 0.3
17	Ellagic acid deoxyhexoside	4.1	447.0568	**301.00**	C_20_H_16_O_12_	−0.2	0.45 ± 0.03
​	**Total amount (g/100 g dry extract)**	​	​	​	​	​	**13.2 ± 0.9**
Propolis metabolites							
10	Caffeic acid	5.0	179.0347	**135.04**, 117.03, 89.04	C_9_H_8_O_4_	−1.6	0.32 ± 0.05
12	*p*-Coumaric acid	7.8	163.0395	**119.05**, 93.03, 65.04	C_9_H_8_O_3_	−3.5	-
18	Quercetin	16.2	301.0353	257.82, 193.81	C_15_H_10_O_7_	−0.3	0.09 ± 0.01
19	Pinobanksin 5-methyl ether	17.9	285.0766	267.07, 252.04, 239.07	C_16_H_14_O_5_	−0.9	0.72 ± 0.05
20	Quercetin methyl ether I	18.0	315.0509	**300.03**, 271.02, 255.03, 151.00	C_16_H_12_O_7_	−0.4	0.36 ± 0.02
21	Pinobanksin^e^	18.2	271.0611	253.05, 243.07, 225.06, 151.00	C_15_H_12_O_5_	−0.4	0.82 ± 0.04
22	Apigenin	18.9	269.0455	225.90, 197.81, 151.00	C_15_H_10_O_5_	−0.2	0.72 ± 0.06
23	Quercetin methyl ether II	19.5	315.0511	**300.03**, 271.02, 255.03, 151.00	C_16_H_12_O_7_	+0.2	0.25 ± 0.02
24	Luteolin methyl ether I	20.0	299.0558	**284.03**, 255.03	C_16_H_12_O_6_	−1.0	0.27 ± 0.01
25, 26	Caffeic acid prenyl ester I and II	21.0, 21.5	247.0972	179.03, 135.04	C_14_H_16_O_4_	−1.5	9.8 ± 0.8
27, 28	Caffeic acid benzyl ester I and II	22.1, 22.3	269.0817	137.02, 134.03	C_16_H_14_O_4_	−0.9	2.2 ± 0.4
29	Quercetin methyl ether III	22.6	315.0510	**300.03**, 271.02, 256.04, 193.01, 165.02	C_16_H_12_O_7_	−0.1	0.31 ± 0.02
30, 31	*p*-Coumaric acid prenyl ester I and II	22.9	231.1022	163.04, 119.05	C_14_H_16_O_3_	−2.0	-
32	Galangin^e^	23.2	269.0453	227.03, 197.06	C_15_H_10_O_5_	−0.9	0.46 ± 0.03
33	Caffeic acid phenylethyl ester (CAPE)^e^	23.4	283.0972	179.03, 135.04	C_17_H_16_O_4_	−1.3	2.3 ± 0.4
34	Pinocembrin^e^	23.6	255.0661	213.06, 211.08, 151.00	C_15_H_12_O_4_	−0.7	1.6 ± 0.1
35	Chrysin	23.8	253.0504	209.06, 167.05, 151.00, 143.05	C_15_H_10_O_4_	−0.9	2.9 ± 0.2
36	Pinobanksin 3-*O-*acetate	24.0	313.0717	271.06, 253.05, 209.06	C_17_H_14_O_6_	−0.2	2.5 ± 0.1
37	Luteolin methyl ether II	24.5	299.0560	284.03, 256.04, 255.06, 151.00	C_16_H_12_O_6_	−0.4	0.11 ± 0.01
38	Galangin methyl ether	24.8	283.0610	268.04, 239.03, 211.04	C_16_H_12_O_5_	−0.7	0.79 ± 0.08
39	Pinostrobin	25.2	269.0819	254.06, 251.07, 178.03, 165.02	C_16_H_14_O_4_	−0.1	0.63 ± 0.05
40	Caffeic acid cinnamoyl ester	25.3	295.0974	251.11, 211.08, 178.03, 134.04	C_18_H_16_O_4_	−0.6	-
41	Pinobanksin 3-*O*-propionate	25.8	327.0872	271.06, 253.05	C_18_H_16_O_6_	−0.6	0.75 ± 0.05
42	Pinobanksin 3-*O*-phenylpropionate	26.3	403.1186	271.06, 253.05	C_24_H_20_O_6_	−0.3	0.030 ± 0.004
43	Pinobanksin 3-*O*-butyrate	27.0	341.1026	271.06, 253.05	C_19_H_18_O_6_	−1.3	0.56 ± 0.01
44	Pinobanksin butyrate isomer	27.2	341.1028	271.29, 253.05	C_19_H_18_O_6_	−0.8	0.034 ± 0.002
45	Caffeic acid derivative	27.9	399.2172	179.03, 135.04	C_24_H_32_O_5_	−1.3	-
46	Pinobanksin 3-*O*-pentanoate	28.3	355.1183	271.06, 253.05	C_20_H_19_O_6_	−1.1	0.71 ± 0.04
47	Caffeic acid derivative	28.5	413.1966	251.17, 179.03, **161.02**, 133.03	-	-	-
48	Fatty acid	28.9	293.2120	249.22, 185.12, 125.10	C_18_H_30_O_3_	−0.7	​
[M + H]^**+**^							
49	3,4-Dimethyl caffeic acid (DMCA)	16.4	209.0807	191.07, 163.07, 148.05, 133.03	C_11_H_14_O_4_	0	-
50	Quercetin dimethyl ether I	21.1	331.0810	316.06, 302.04	C_17_H_14_O_7_	−0.3	0.50 ± 0.04
51	Chrysin methyl ether I	21.6	269.0807	254.06	C_16_H_12_O_4_	−0.4	0.31 ± 0.01
52	Quercetin dimethyl ether II	24.1	331.0811	316.06, 301.03	C_17_H_14_O_7_	−0.3	1.3 ± 0.1
53	Alpinetin	28.2	271.0962	167.03, 131.05, 103.05	C_16_H_14_O_4_	−0.7	1.6 ± 0.1
54	Chrysin methyl ether II	28.6	269.0807	254.06	C_16_H_12_O_4_	−0.4	0.99 ± 0.07
Total propolis metabolites (g/100 g dry extract)			​	​	​	​	**33.9 ± 2.8**
Total constituents Pp1:2 preparation (g/100 g dry extract)			​	​	​	​	**47.1 ± 3.7**

^a^ Compound numbers correspond with the peak numbers in Figure 2.

^b^ Product ions are generated by the fragmentation of [M − H]^-^

or [M + H]^+^; the base ion peaks generated in the ESI-MS/MS, experiments are shown in bold.

^c^ Doubly charged ion species [M - 2H]2‒

generated by the molecule at 1084 u.

^d^ The amount was calculated by LC-MS, quantitative analyses constructing calibration curves using reference standard.

^e^ Confirmed by injection of reference standards.

DHHDP, dehydrohexahydroxydiphenic acid; HHDP, bis-hexahydroxydiphenoyl hexoside.

As expected, Pp1:2 was rich in chemical bioactive agents, belonging to different classes of specialized metabolites. Pomegranate peels were confirmed as a source of polyphenols, including gallic acid (**3**), ellagic acid (**16**) and its glycosides (**13**, **15**, and **17**), ellagitannins with punicalagin (isomers **6** and **11**) and pedunculagin (isomers **7** and **9**), known as the major hydrolyzable tannins of pomegranate fruit (Singh et al., 2018). Propolis contributed to enrich the preparation of other classes of specialized metabolites, represented mostly by flavonoids and phenolic acids. Due to hydrolysis of glycosidic linkages induced by bees through their glycosidase salivary enzymes, only flavonoid aglycones were detected as propolis metabolites. As reported in our previous! study concerning Italian propolis from Campania region (Parisi et al., 2020), flavones such as apigenin (**22**), luteolin methyl ether (**24** and **37**), galangin (**32**), and chrysin (**35**), flavanones including pinocembrin (**34**) and alpinetin (**53**), the flavonol quercetin (**18**), and the dihydroflavonols pinobanksin (**19**) were found. Moreover, methoxylated flavonoids were detected, while pinobanksin occurred also in esterified form with acetate (**36**), propionate (**41**), phenylpropionate (**42**), butyrate (**43**), and pentanoate (**46**). Caffeic acid (**10**) and its prenyl (**25** and **26** isomers), benzyl (**27** and **28** isomers), phenylethyl (CAPE, **33**), and cinnamoyl esters (**40**) were identified in the Pp1:2 preparations. Finally, *p*-coumaric acid (**12**) and its prenyl esters (isomers **30** and **31**) were found.

Quantitative analysis of the identified compounds (Table 2) showed that 100 g of the dry preparation contained 47.1 ± 3.7 g of polyphenols. Of these, 13.2 ± 0.9 g were from pomegranate peel while 33.9 ± 2.8 g from propolis. In agreement with the literature (Singh et al., 2018), the most abundant pomegranate phytochemicals were ellagic acid (**16**, 4.8 ± 0.3 g/100 g), punicalagin isomers (**6** and **7**, 1.8 ± 0.9 g/100 g), HHDP-hexoside (**2**, 1.6 ± 0.1 g/100 g), and gallic acid (**3**, 1.4 ± 0.1 g/100 g). Propolis enriched the preparation mainly in caffeic acid esters, with caffeic acid prenyl ester (**25** and **26** isomers) reaching 9.8 ± 0.8 g/100 g, and CAPE (**33**) and caffeic acid benzyl ester (**27** and **28** isomers), which were in similar amount (2.3 ± 0.4 g and 2.2 ± 0.4 g/100 g, respectively). Among flavonoid, chrysin (**35**, 2.9 ± 0.2 g/100 g) and its methyl ether isomers (**51** and **54**) were found in a great amount; also pinobanksin (**31**) and its derivatives significantly contributed to the phenol content of preparation, being pinobanksin 3-*O*-acetate (**36**, 2.5 ± 0.1 g/100 g) the most represented pinobanksin-related compound. Finally, pinocembrin (**34,** 1.6 ± 0.08 g/100 g), alpinetin (**53**, 1.6 ± 0.06 g/100 g), and galangin (**32** and its methoxy derivative **38**) were included among the major propolis-derived constituents of the preparation. These results were comparable to previous analyses of Italian propolis collected in different regions, suggesting the use of similar botanical source plants by bees for Italian propolis production. CAPE, chrysin, pinobanksin, pinocembrin, and galangin could be considered potential markers of Italian propolis (Pellati et al., 2011; De Zordi et al., 2014; Miraldi et al., 2024).

### 3.3 Pp1:2 inhibits M1 macrophage polarization in human THP-1 cells

Since the balance between M1 and M2 macrophage phenotypes is a key driver of RA synovitis (Cutolo et al., 2022), we preliminarily investigated whether Pp1:2 could modulate macrophage polarization. Using PMA-differentiated THP-1 macrophages stimulated with LPS and IFNγ to induce a proinflammatory M1-like phenotype, we evaluated the expression of the specific M1 marker CD86. Immunofluorescence analysis revealed that co-treatment with Pp1:2 significantly suppressed LPS/IFNγ-induced CD86 expression compared to cells stimulated with LPS/IFNγ alone (Figure 3), thereby demonstrating a robust inhibitory effect on M1 macrophage polarization.

**FIGURE 3:**
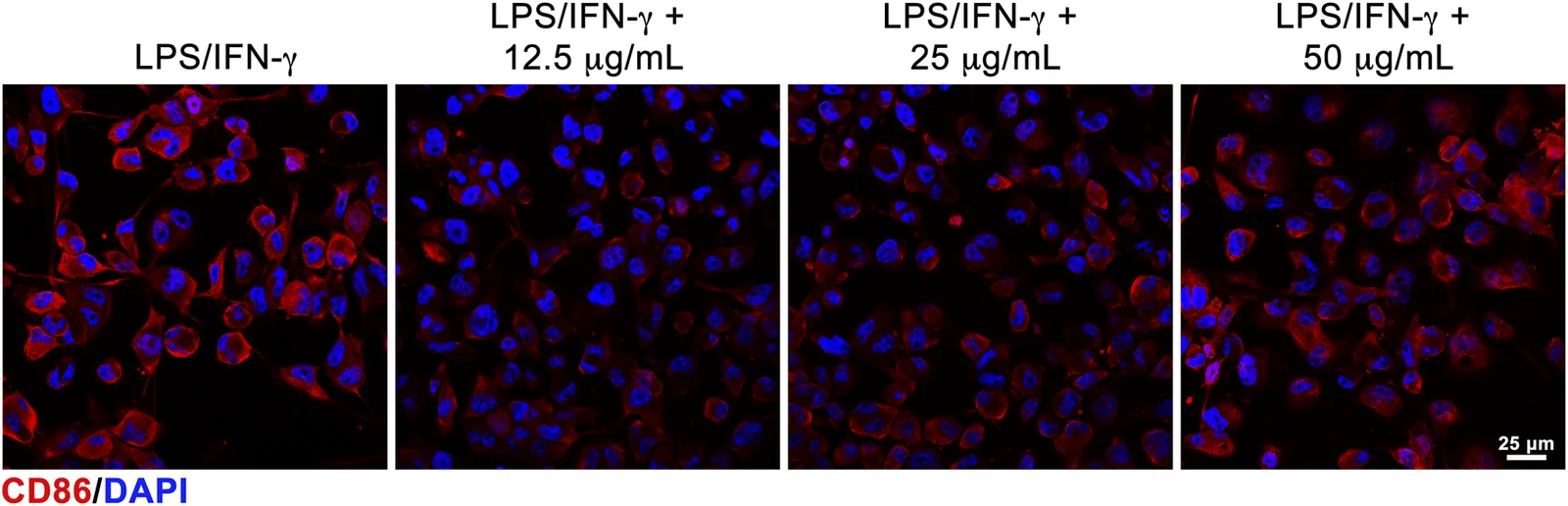
Pp1:2 attenuates M1 polarization in THP-1 cells. Representative fluorescence images of CD86 (red) in LPS/IFNγ-treated THP-1 cells. DAPI (blue) was used to stain nuclei.

### 3.4 Pp1:2 treatment induces cytokine modulation on human IL-1β-stimulated synoviocytes

Synovial cells play a major role in the pathophysiology of RA and have recently been identified as an important target for anti-RA therapies (Svensson et al., 2020). Therefore, we assessed the anti-inflammatory activity of the preparation using human SW982 synoviocytes (Chang et al., 2014) stimulated with IL-1β. In that aim, we evaluated the changes in secretion of the main interleukins, IL-6 and IL-8, induced by SW982 incubation with different concentrations of Pp1:2 (12.5, 25, and 50 μg/mL). Preliminarily, we confirmed that these incubation conditions had negligible effects on SW982 cells viability (Supplementary Figure S1). As shown in Figures 4A,B, Pp1:2 is able to induce a reduction in the secretion of both cytokines in a dose-dependent manner. At the highest dose of 50 μg/mL, Pp1:2 induced a reduction of about 60% particularly for IL-8 and even higher for IL-6.

**FIGURE 4:**
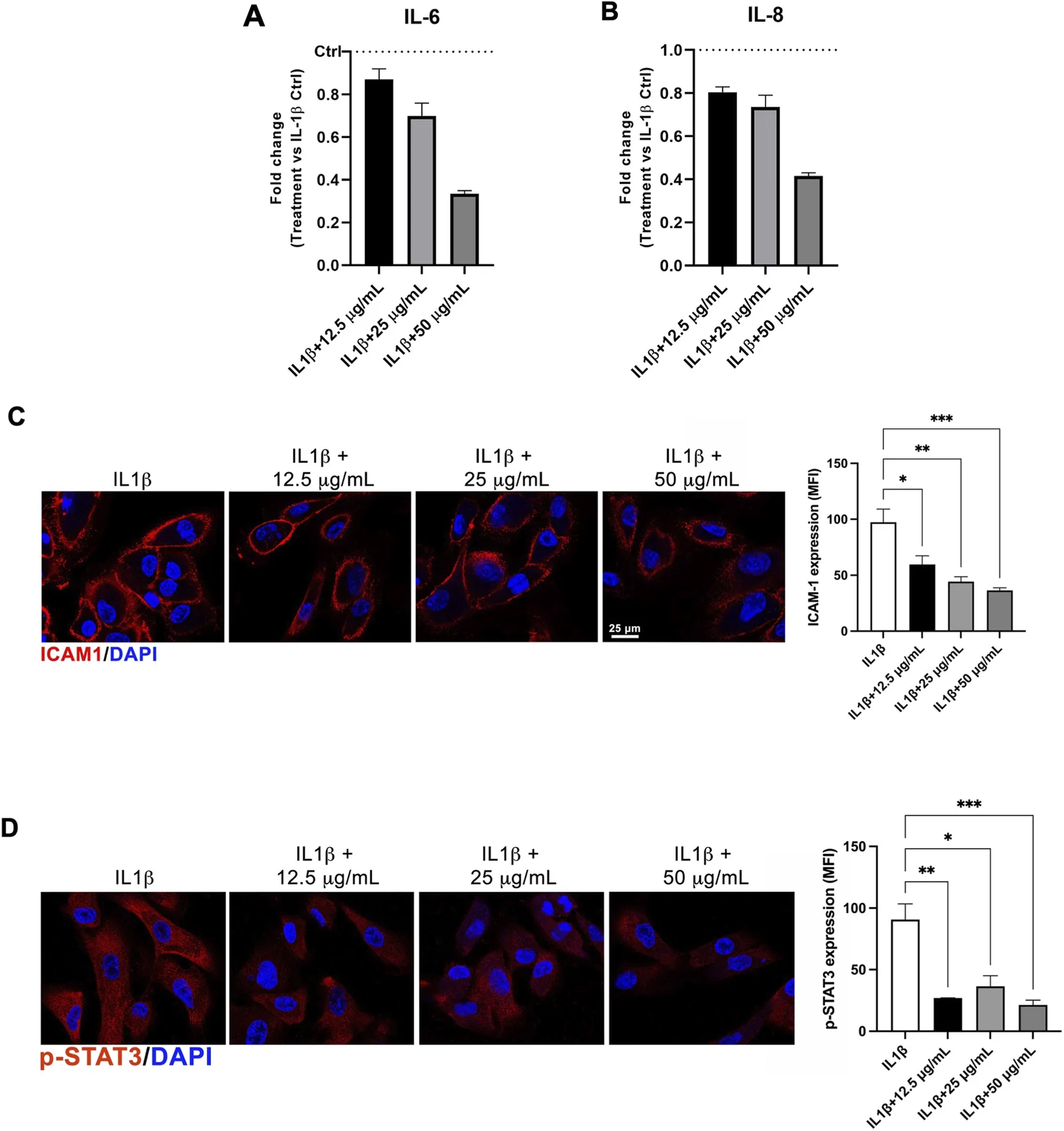
Anti-inflammatory effects of Pp1:2 evaluated on IL-1β-stimulated synoviocytes. IL-6 **(A)** and IL-8 **(B)** modulation in response to the treatment with Pp 1:2 treatment at doses of 12.5, 25 and 50 μg/mL on IL-1β-stimulated synoviocytes for 3 h. Representative fluorescence images of IL-1β-stimulated synoviocytes immunostained for ICAM-1 **(C)** and phosphorylated STAT3 **(D)**. DAPI was used to counterstain nuclei (blue). Bar graphs showing mean fluorescence intensity (MFI) quantification (arbitrary units) **(C,D)**. One-way ANOVA with Tukey *post hoc* comparison was used for comparisons between groups. *p = 0.01; **p = 0.002; ***p < 0.001 **(C,D)**.

Intercellular Adhesion Molecule-1 (ICAM-1) plays a pivotal role in leukocyte recruitment to the inflamed synovium (Koch et al., 1991), acting as a critical target for therapeutic modulation in RA (Yusuf-Makagiansar et al., 2002). To further investigate the impact of Pp1:2 on synoviocytes activation, we evaluated the expression of ICAM-1 through immunofluorescence analysis. As expected, stimulation of human SW982 synoviocytes with IL-1β triggered a robust increase in ICAM-1 signal, characterized by an intense membrane fluorescence (Figure 4C). Notably, treatment with Pp1:2 significantly attenuated this effect with a dose-dependent decrease in ICAM-1 staining intensity, confirming that the preparation not only modulates soluble cytokine secretion but also downregulates the expression of key surface adhesion molecules involved in the inflammatory cascade of RA. Signal Transducer and Activator of Transcription 3 (STAT3) is a central regulator in the pathogenesis of RA, and its inhibition has been shown to effectively suppress both joint inflammation and osteoclast activation (Lee et al., 2017; Xu et al., 2024). Given this regulatory role, we further examined whether Pp1:2 modulates this signaling pathway. Immunofluorescence analysis revealed that treatment with Pp1:2 significantly reduced p-STAT3 levels compared to cells stimulated with IL-1β alone (Figure 4D). Altogether, these findings demonstrate that Pp1:2 exerts a broad anti-inflammatory effect, potentially limiting monocyte recruitment and synovial infiltration through the dual downregulation of adhesion molecules and pro-inflammatory signaling intermediates.

### 3.5 Pp1:2 reduces RA related inflammation and symptoms of the pathology *in vivo*

The efficiency of Pp1:2 as an anti-RA agent was evaluated using an *in vivo* model of RA. Arthritis was induced by using collagen from chicken sternal cartilage Type II to 40 females 8–12 weeks old DBA1/J mice, which were divided in 8 groups according to the different treatments (Supplementary Table S1). To confirm that the effective intake of bioactive compounds from Pp1:2, the concentration of some of the most representative components of the preparation was measured in plasma of mice (Supplementary Figure S2) 30 min and 60 min after oral administration of subjected to the administration of 150 mg/kg Pp1:2. In accordance with the chemical profile of the preparation previously described, propolis metabolites dominated in the serum. Specifically, four phenols derived from propolis (chrysin, galangin, pinocembrin, and CAPE) were positively detected. Interestingly, pinocembrin was found to be in a higher amount than chrysin, even if the latter was more abundant in the preparation. Instead, ellagic acid provided by pomegranate peel was not detected at all in any sample, probably due to its very low oral bioavailability. Urolithins are reported as the major metabolites of both ellagitannins and ellagic acid produced by gut microbiota, being urolithin A the most bioavailable molecule (Yang et al., 2024). In the analysed serum samples, urolithin A was detected indicating the absorption of ellagic acid/ellagitannins; however its quantification was not performed because the concentration values were <LOQ. This result was quite expected, since the production of these metabolites strongly depends on the presence of urolithin-producing bacterial strains that could not be found in all individuals (Yang et al., 2024). Therefore, the concentration of urolithins in biological fluids is affected by a high variability, maybe due to the source and/or dosage or analytical method used for quantification. Besides, differences were found in rats, mice, and humans (Lin et al., 2023).

These results confirmed the gastro-intestinal absorption of most of the components of the Pp1:2 preparation, further demonstrating that metabolic processes did not lead to a complete modification or elimination of these compounds. The measurement of body weight carried out weekly on all mice demonstrated that the treatment did not induce significant effects on this parameter, regardless of the dose administered (Supplementary Figure S3).

The progress of RA was assessed twice a week by observing the characteristic symptoms of arthritis (edema and swelling) on the paws of the mice, and consequently assigning a score to each animal on a scale from 0 to 4. Changes in paw volume were recorded weekly. The most promising results were obtained in mice receiving 150 mg/kg Pp1:2, administered either prior to (preventive, GP4) or simultaneously with (curative, GP5) inflammatory induction and the effectiveness of the treatment appeared to reach its maximum after 72 days (Figure 5A). While both the preventive and curative 150 mg/kg-treated groups showed a greater paw volume reduction compared to the vehicle-treated group, the *post hoc* trend analysis revealed no statistically significant linear dose-dependency across the three dose levels. In more detail, a statistically significant increase (p < 0.0001-p<0.0002) in paw thickness for both forelimbs and hindlimbs in the CIA-induced groups compared to the non-induced control group was measured (data not shown), which was time dependently reduced by preventive and curative treatment with 150 mg/kg Pp1:2, although the anti-inflammatory action was clearly more effective for GP5 animals (Figures 5B–D). This anti-inflammatory effect was further confirmed by analyzing the levels of several cytokines, including IL-6, IL-1β, and IL-17 A (Supplementary Tables S2, S3). Among these, the data regarding the cytokine IL-17 A showed the most promising results in response to treatment after 79 days, particularly for the curative treatment (Figure 5D). IL-6 and IL-1β were not detectable according to the manufacturer’s instructions of ELISA assay (Supplementary Table S4). These clinical observations were validated by histological analysis. Safranin-O staining demonstrated that the curative administration of 150 mg/kg Pp1:2 more effectively preserved proteoglycan content and attenuated cartilage erosion compared to untreated CIA mice, thereby corroborating the protective properties of the mixture (Figure 5E).

**FIGURE 5:**
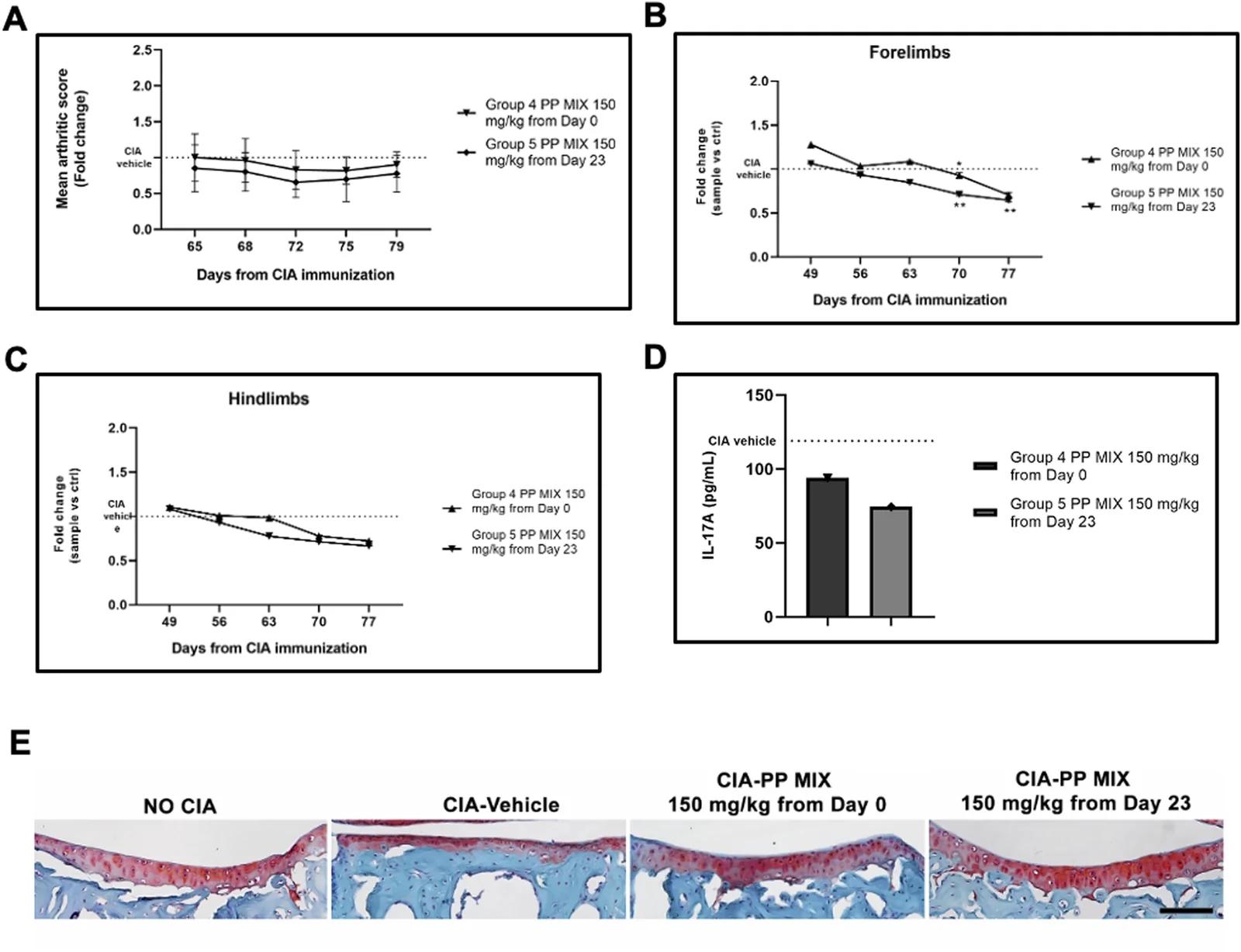
Anti-inflammatory effects of Pp1:2 evaluated in a mouse model of RA. Average arthritic score measure after 65, 68, 72, 75, and 79 days of treatment for mice group subjected preventive (GP4) or therapeutic (GP5) administration of 150 mg/kg of Pp1:2 **(A)**. Average paw volumes of forelimbs (FL) and hindlimbs (HL) measured after 56, 63, 70, and 77 days of treatment for mice group subjected preventive (GP4) or therapeutic (GP5) administration of 150 mg/kg of Pp1:2 **(B,C)**. IL-17 A measurement after 79 days of treatment for mice group subjected preventive (GP4) or therapeutic (GP5) administration of 150 mg/kg of Pp1:2 **(D)**. Representative micrographs of Safranin O-stained hind paws sections across experimental groups; red intensity indicates proteoglycan content within the articular cartilage. Scale bar: 100 µm **(E)**. One-way ANOVA with Tukey *post hoc* comparison was used for comparisons between groups. *p < 0.05; **p < 0.01 **(A–C)**.

### 3.6 Pp1:2 treatment induces M1-to-M2 macrophage polarization within the synovial tissues of CIA mice

To elucidate the immunomodulatory mechanisms of the formulation, we assessed the expression of CD68 (a pan-macrophage marker reflecting total macrophage infiltration) and CD163 (a well-established marker for the anti-inflammatory M2 macrophage phenotype) within the inflamed paw joints. As expected, CD68 expression was markedly elevated in the CIA-vehicle group (Figure 6A). Given that early-stage joint inflammation in this model is heavily driven by a massive influx of pro-inflammatory, M1-polarized macrophages, this high baseline of total macrophage infiltration aligned with the progression of joint destruction (Chen et al., 2025). Conversely, administration of 150 mg/kg Pp1:2 significantly suppressed CD68 levels in the synovial tissues compared to the vehicle-treated group. Parallel to this downregulation, both the preventive and curative paradigms effectively upregulated CD163 expression (Figure 6A). To confirm the effect of Pp1:2 on macrophage polarization, immunofluorescence staining was conducted to assess the expression of the specific M1 macrophage marker inducible Nitric Oxide Synthase (iNOS). iNOS expression was increased in untreated CIA mice, whereas Pp1:2 significantly reduced iNOS-positive staining in both treated groups (Figure 6B).

**FIGURE 6:**
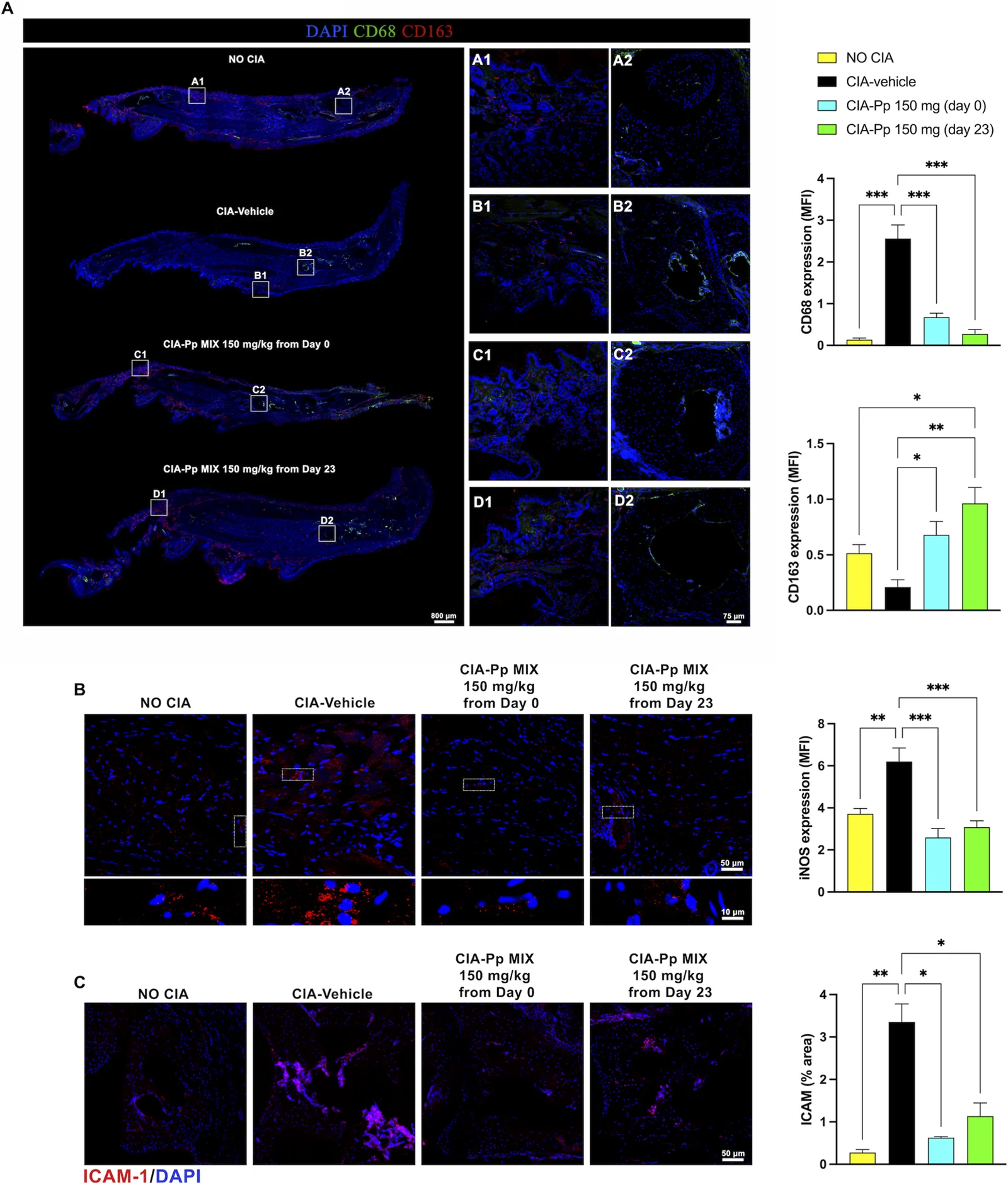
Immunomodulatory effects of Pp1:2 in CIA-induced hind paws. Representative immunofluorescence images and relative magnification insets **(A–C)** of CD68 (green) and CD163 (red) in joint tissue from the hind paws of each experimental group. DAPI (blue) was used to stain nuclei. Bar graphs showing mean fluorescence intensity (MFI) quantification (arbitrary units) of CD68 and CD163 **(A)**. Representative immunofluorescence images and relative magnification insets of iNOS (red) and nuclei (DAPI, blue) in hind paw joint tissue across experimental groups. Bar graph showing mean fluorescence intensity (MFI) quantification (arbitrary units) **(B)**. Representative immunofluorescence images of ICAM-1 (red) and nuclei (DAPI, blue). Bar graph showing quantification of ICAM-1 positive staining area in paw sections **(C)**. One-way ANOVA with Bonferroni *post hoc* comparison was used for comparisons between groups. *p < 0.05; **p = 0.001; ***p < 0.001 **(A)**. *p < 0.05; **p < 0.01 **(B,C)**.

Taken together, these findings indicate that Pp1:2 treatment promotes a favorable phenotypic shift in the macrophage population, transitioning from an arthritogenic, pro-inflammatory M1 state toward a protective, anti-inflammatory M2 phenotype. This result is consistent with previous reports demonstrating that CD163 deficiency exacerbates disease severity in murine CIA models (Svendsen et al., 2020). Furthermore, Pp1:2 administration significantly reduced ICAM-1 expression in the hind paws of CIA mice compared to untreated controls and attenuated the pathological remodeling of collagen IV (Figure 6C; Supplementary Figure S4).

### 3.7 Study limitation

Our research has provided encouraging data on the efficacy of Pp1:2 in reducing the effects of RA *in vivo*, but it is important to highlight some limitations of this study. First, it is important to emphasize that the aim of this study was to confirm, through complementary *in vivo* and *in vitro* studies, the actual anti-inflammatory potential of the mixture under investigation in RA models, while also providing information on the molecular mechanisms involved in this effect. Therefore, determining whether the results obtained can be extrapolated to humans will require further studies specifically designed for this purpose. Indeed although mouse models are widely used to investigate the effects of anti-inflammatory agents, there are obvious issues related to species differences (Firestein, 2009), including the inevitable approximations regarding the extrapolation of the dosage to be used in humans. This last point is made even more challenging by the great chemical heterogeneity of the mixture we studied; in this case, in fact, the response observed as a function of the dosage used cannot be easily predictable or deducible from a limited number of measurements (Srivastava et al., 2025; Wang and Wang, 2025). Besides, although animals receiving Pp1:2 exhibited no overt signs of physical toxicity or weight loss during the experimental period, we have no data on specific biomarkers for renal and hepatic toxicity.

Furthermore, since this study was based on the traditional use of certain preparations from rural areas of southern Italy, we used only propolis and pomegranates from those regions. Given that the inherent variability of the secondary metabolites in these natural products closely correlates with their region of production, this limits the generalizability of the results we obtained. It should be emphasized, however, that a detailed description of the chemical composition of Pp1:2 would make it possible to obtain similar preparations even from propolis and pomegranates of different origins.

Finally, in light of the study’s objectives mentioned above and the complexity of the mixture used, we did not use an appropriate positive control in the various tests. This precludes any quantitative comparison between the anti-inflammatory efficacy of Pp1:2 and that of drugs typically used to treat patients with RA. Indeed, it would have made little sense to compare the effects of a pure molecule—with a specific mechanism of action used for therapeutic purposes—with a heterogeneous mixture that is presumably capable of acting on multiple targets and that could be used, once the necessary studies are completed, as a palliative treatment to reduce the symptoms of inflammation in patients with RA.

## 4 Discussion

Rheumatoid arthritis is a chronic inflammatory disease associated with autoimmunity, whose etiology is not entirely clear, nor is its diagnosis, due to its multifactorial nature (Radu and Bungau, 2021). Moreover, diagnosis is often complicated and frequently based on symptoms rather than on more specific clinical evidence (Cush, 2022). Treating patients affected by RA is therefore difficult and often involves the use of medications that only partially resolve the problem. Furthermore, given the chronic nature of the disease, many treatments may lose their effectiveness over time or cause side effects, some of which can be severe (Del Grossi Moura et al., 2018). Based on this evidence, in recent years several treatments exploiting natural compounds have been proposed (Moudgil and Venkatesha, 2022). In many cases, the key factor determining the high efficacy of these preparations is their complex composition, which combines numerous molecules with additive, complementary, and synergistic effects (Hosseini et al., 2018; Barros et al., 2021). This aspect is particularly important specifically for the treatment of multifactorial inflammatory conditions such as RA. However, for a preparation based on natural substances to be effectively considered as an adjunct in the treatment of RA, its effects must be evaluated by providing information at the molecular level as well, also because, given the chemical complexity of certain formulations, the risk of adverse effects should not be underestimated (Anywar et al., 2021; Belorio and Gómez, 2022).

The aim of the present study was to evaluate the efficacy of preparations containing propolis and pomegranate juice or peel hydroalcoholic extract as anti-inflammatory agents for the treatment of patients with RA. A first not-secondary finding emerged from the preliminary screening conducted on various preparations to identify those with the greatest ability to suppress inflammation in macrophage cells. This study demonstrated that by macerating pomegranate peels—which constitute approximately 40%–50% of the fruit and are a byproduct of the agri-food industry—in a hydroalcoholic solution, it is possible to obtain an extract with anti-inflammatory properties comparable to those of the juice, taking into account the obvious differences in the amount to be used. This finding was further confirmed by the chemical characterization of the Pp1:2 preparation, in which numerous bioactive metabolites typical of pomegranate juice were identified in significant concentrations. Moreover, previous studies had already highlighted the richness of pomegranate peels in secondary metabolites, mainly emphasizing their nutritional and antioxidant potential (Ain et al., 2023). Therefore, considering the absolute biocompatibility of the extraction procedure used and its low cost, it is clear that pomegranate peels represent a resource that should not be wasted. The results of the initial screening also reveal another interesting insight. Observing the anti-inflammatory activity of the various preparations tested reveals that the propolis-to-pomegranate peel extract ratio plays a critical role. This finding highlights once again that the great heterogeneity of phytocomplexes requires careful evaluation of the qualitative and quantitative composition of the various bioactive molecules to ensure balance among the different possible effects.

With regard to the evaluation of the biological effect of Pp1:2, the results obtained in various RA models are consistent and suggest that the preparation acts by interfering with several key pathogenic mechanisms. A crucial finding is the ability of Pp1:2 to inhibit pro-inflammatory M1 macrophage polarization. Given that an imbalance in macrophage polarization—characterized by a shift toward the pro-inflammatory M1 phenotype over the protective M2 phenotype—serves as a primary pathogenic driver of synovial inflammation and subsequent bone erosion in RA (Cutolo et al., 2022), this modulation suggests that the preparation could effectively counteract the chronic inflammatory environment characteristic of the disease. This finding is strongly corroborated by our *in vivo* results. Beyond the significant reduction in clinical markers of arthritis, we observed a decrease in the pro-inflammatory M1 population and an increase in the protective M2 population in Pp1:2-treated CIA mice, similar to what was previously observed for pomegranate juice (Aharoni et al., 2015) and pure punicalagin (Ge et al., 2022). This Pp1:2-induced shift in the macrophage population may also underlie the decrease in ICAM-1 levels observed in the paws of animals treated with the preparation. In fact, the cross-talk between the abundance of this protein in inflamed tissues and the M1/M2 macrophage phenotype ratio has been well established (Qian et al., 2024).

The present study further demonstrates that Pp1:2 exerts anti-inflammatory effects in RA models by targeting non-redundant molecular pathways. Given the key role of synoviocytes in the pathophysiology of RA (Svensson et al., 2020), we evaluated the effect of the mixture on these cells following stimulation with IL-1β and we observed that Pp1:2 significantly attenuates the secretion of major pro-inflammatory cytokines, IL-6 and IL-8. This finding is of particular clinical relevance, as these interleukins are primary drivers of inflammation and cartilage destruction (Chang et al., 2014). Moving beyond soluble mediators, our data reveal that Pp1:2 decreases ICAM-1 surface expression and attenuates the phosphorylation of STAT3. This reduction suggests that Pp1:2 may limit the infiltration of inflammatory cells into joint tissues. ICAM-1 is a cornerstone of the inflammatory cascade, mediating the leukocyte recruitment into the inflamed synovium, and its targeted inhibition is increasingly recognized as a therapeutic strategy to prevent synovial hyperplasia (Koch et al., 1991; Yusuf-Makagiansar et al., 2002). Concurrently, STAT3 serves as a central node in promoting sustained inflammation and joint destruction, particularly through its role in the IL-1β and TNFα-initiated IL-6–STAT3 signaling axis, which drives both inflammatory cytokine production and RANKL-mediated osteoclastogenesis (Mori et al., 2011; Lee et al., 2017). Moreover, recent evidence highlights that STAT3 activation directly regulates the expression of adhesion molecules, including ICAM-1, across several inflammatory conditions (Wang et al., 2021), thereby potentially creating a feed-forward loop that sustains chronic joint inflammation and promotes bone erosion. By dually downregulating these adhesion molecules and pro-inflammatory signaling intermediates, Pp1:2 exerts a broad anti-inflammatory effect that likely limits both monocyte recruitment and synovial infiltration.

The complexity of Pp1:2 chemical composition prevents the conduct of a thorough study aimed at demonstrating the exact mechanism underlying the various observed effects. However, the results of analyses conducted by monitoring various molecular markers, together with the efficacy of the preparation observed *in vivo* following oral administration, make Pp1:2 a promising candidate for an adjuvant non-pharmacological treatment of the inflammation-related symptoms in RA patients. From this perspective, the results obtained in studies using a mouse model could also make it possible to evaluate a potential dosage for use in humans. Indeed, using body surface area (BSA) normalization, the effective preclinical murine can be converted to a human equivalent dose (HED) of 12.16 mg/kg, which falls well within the therapeutic ranges independently established as safe and effective in human clinical settings for both individual compounds. Specifically, standardized propolis extracts have been safely administered in randomized controlled trials at dosages ranging from 200 to 1000 mg/day to modulate inflammatory and oxidative biomarkers (Magnavacca et al., 2022). Similarly, *P. granatum* peel extracts are routinely employed in clinical protocols at oral doses of 400–1000 mg/day to leverage their anti-inflammatory properties (Rafraf et al., 2024). However, although the calculated human dose represents a realistic, safe, and practically achievable therapeutic regimen for future human evaluation, it is important to underline that when dealing with complex mixtures such as Pp1:2, the optimal dose must be determined through specific studies. Indeed, as shown by the results we obtained from the various groups of animals treated with different protocols, the response to the administration of combinations containing many different compounds often does not follow linear and predictable patterns (Srivastava et al., 2025; Wang and Wang, 2025). Besides, the variability in the effects observed when using herbal remedies depending on the administered dose is widely reported (Busia, 2024) and underlies the well-known phenomenon of hormesis (Son et al., 2008). Therefore, in order to translate our result to possible human use, further investigations will be required.

## Data Availability

The data presented in the study are deposited in the Zenodo repository, available at https://doi.org/10.5281/zenodo.22941746
