# Supplementary material for: A mixture of pomegranate peels and propolis extracts reduces inflammation associated with rheumatoid arthritis in vivo
Source: Front Pharmacol. 2026 Oct 7;17:1910652. doi: 10.3389/fphar.2026.1910652 (PMC13683642; doi:10.3389/fphar.2026.1910652)
Supplement: Supplementary file 1 [file Table1.docx]

**Table S1** Description of animal groups and respective treatments.

| **Experimental Group** | **Sex** | **N° of animals** | **ID Animals From-To** | **CIA** | **Treatment** | **Dose**  **(mg/kg)** | **Route** | **Schedule** |
| --- | --- | --- | --- | --- | --- | --- | --- | --- |
| GP1 | F | 5 | 1 – 5 | YES | VEHICLE | - | OS gavage | daily from day 0 |
| GP2 | F | 5 | 6 – 10 | YES | PP | 100 | OS gavage | daily from day 0 |
| GP3 | F | 5 | 11 – 15 | YES | PP | 100 | OS gavage | daily starting from day +23 |
| GP4 | F | 5 | 16 - 20 | YES | PP | 150 | OS gavage | daily from day 0 |
| GP5 | F | 5 | 21 – 25 | YES | PP | 150 | OS gavage | daily starting from day +23 |
| GP6 | F | 5 | 26 - 30 | YES | PP | 200 | OS gavage | daily from day 0 |
| GP7 | F | 5 | 31 – 35 | YES | PP | 200 | OS gavage | daily starting from day +23 |
| GP8 | F | 5 | 36 - 40 | NO | - | - | - |  |

**Table S2. Levels of IL-6 measured in the experimental cohort.** Columns: Experimental group (GP), Treatment type, Animal ID, vial’s label, mean of OD 450nm values, CV%, mean of IL-6 concentration measured in the assay, Dilution Factor (DF), and IL-6 concentration per mL of serum. BLOD, below the Limit of Detection. In red, values below the lower limit of quantitation (LLOQ).

| **Group** | **Treatment** | **ID** | **Tube Label** | **Average OD450nm** | **CV%** | **Average Interpolated [IL-6], pg/mL** | **DF** | **IL-6,**  **pg/mL** |
| --- | --- | --- | --- | --- | --- | --- | --- | --- |
| GP1 | Vehicle | 1 | B16/P/AL2/1 | 0.122 | 5% | 5.67 | 10.0 | 56.67 |
|  |  | 2 | **B16/P/AL2/2** | 0.558 | 2% | 314.83 | **16.7** | 5.247.10 |
|  |  | 4 | B16/P/AL2/4 | 0.105 | 3% | - | 10.0 | BLOD |
|  |  | 5 | B16/P/AL1/5 | 0.085 | 4% | - | 10.0 | BLOD |
| GP2 | PP MIX. 100mg/Kg (from D0) | 6 | B16/P0'/AL2/6 | 0.118 | 4% | - | 10.0 | BLOD |
|  |  | 7 | B16/P0'/AL2/7 | 0.099 | 1% | - | 10.0 | BLOD |
|  |  | 8 | B16/P30'/AL2/8 | 0.191 | 1% | 82.28 | 10.0 | 822.77 |
|  |  | 9 | B16/P30'/AL2/9 | 0.107 | 1% | - | 10.0 | BLOD |
| GP3 | PP MIX. 100mg/Kg (from D23) | 11 | B16/P60'/AL2/11 | 0.103 | 4% | - | 10.0 | BLOD |
|  |  | 12 | B16/P120'/AL2/12 | 0.108 | 1% | - | 10.0 | BLOD |
|  |  | 14 | B16/P240'/AL1/14 | 0.093 | 3% | - | 10.0 | BLOD |
| GP4 | PP MIX. 150mg/Kg (from D0) | 16 | B16/P0'/AL2/16 | 0.150 | 0% | 44.12 | 10.0 | 441.20 |
|  |  | 17 | B16/P0'/AL2/17 | 0.094 | 3% | - | 10.0 | BLOD |
|  |  | 18 | B16/P30'/AL1/18 | 0.092 | 0% | - | 10.0 | BLOD |
|  |  | 19 | B16/P30'/AL1/19 | 0.163 | 5% | 56.92 | 10.0 | 569.15 |
|  |  | 20 | B16/P60'/AL2/20 | 0.091 | 1% | - | 10.0 | BLOD |
| GP5 | PP MIX. 150mg/Kg (from D23) | 22 | B16/P120'/AL2/22 | 0.083 | 6% | - | 10.0 | BLOD |
|  |  | 24 | B16/P240'/AL1/24 | 0.085 | 2% | - | 10.0 | BLOD |
|  |  | 25 | B16/P240'/AL2/25 | 0.092 | 2% | - | 10.0 | BLOD |
| GP6 | PP MIX. 200mg/Kg (from D0) | 26 | B16/P0'/AL2/26 | 0.080 | 6% | - | 10.0 | BLOD |
|  |  | 27 | **B16/P0'/AL2/27** | 0.141 | 3% | 33.02 | **14.3** | 471.74 |
|  |  | 28 | **B16/P30'/AL2/28** | 0.106 | 0% | - | **12.5** | BLOD |
|  |  | 29 | **B16/P30'/AL2/29** | 0.081 | 3% | - | **14.3** | BLOD |
|  |  | 30 | B16/P60'/AL2/30 | 0.088 | 2% | - | 10.0 | BLOD |
| GP7 | PP MIX. 200mg/Kg (from D23) | 31 | B16/P60'/AL1/31 | 0.163 | 5% | 56.92 | 10.0 | 569.15 |
|  |  | 32 | B16/P120'/AL1/32 | 0.126 | 6% | 10.02 | 10.0 | 100.21 |
|  |  | 33 | **B16/P120'/AL2/33** | 0.126 | 3% | 9.58 | **11.1** | 106.45 |
|  |  | 34 | **B16/P240'/AL2/34** | 0.102 | 5% | - | **11.1** | BLOD |
|  |  | 35 | B16/P240'/AL2/35 | 0.089 | 17% | - | 10.0 | BLOD |
| GP8 | CONTROL | 37 | **B16/P/AL1/37** | 0.089 | 8% | - | **12.5** | BLOD |
|  |  | 39 | B16/P/AL2/39 | 0.082 | 8% | - | 10.0 | BLOD |
|  |  | 40 | B16/P/AL2/40 | 0.098 | 2% | - | 10.0 | BLOD |

**Table S3. Levels of IL-1b measured in the experimental cohort.** Columns: Experimental group (GP), Treatment type, Animal ID, vial’s label, mean of OD 450nm values, CV%, mean of IL-1b concentration measured in the assay, Dilution Factor (DF), and IL-6 concentration per mL of serum. BLOD, below the Limit of Detection.

| **Group** | **Treatment** | **ID** | **Tube Label** | **Average OD450nm** | **CV%** | **Average Interpolated [IL-1b ], pg/mL** | **DF** | **IL-1b,**  **pg/mL** |
| --- | --- | --- | --- | --- | --- | --- | --- | --- |
| GP1 | Vehicle | 1 | B16/P/AL2/1 | 0.049 | 0% | - | 10.0 | BLOD |
|  |  | 2 | **B16/P/AL2/2** | 0.049 | 6% | - | **16.7** | BLOD |
|  |  | 4 | B16/P/AL2/4 | 0.047 | 3% | - | 10.0 | BLOD |
|  |  | 5 | B16/P/AL1/5 | 0.046 | 2% | - | 10.0 | BLOD |
| GP2 | PP MIX. 100mg/Kg (from D0) | 6 | B16/P0'/AL2/6 | 0.046 | 6% | - | 10.0 | BLOD |
|  |  | 7 | B16/P0'/AL2/7 | 0.045 | 3% | - | 10.0 | BLOD |
|  |  | 8 | B16/P30'/AL2/8 | 0.050 | 1% | - | 10.0 | BLOD |
|  |  | 9 | B16/P30'/AL2/9 | 0.049 | 1% | - | 10.0 | BLOD |
| GP3 | PP MIX. 100mg/Kg (from D23) | 11 | B16/P60'/AL2/11 | 0.048 | 1% | - | 10.0 | BLOD |
|  |  | 12 | B16/P120'/AL2/12 | 0.046 | 0% | - | 10.0 | BLOD |
|  |  | 14 | B16/P240'/AL1/14 | 0.045 | 6% | - | 10.0 | BLOD |
| GP4 | PP MIX. 150mg/Kg (from D0) | 16 | B16/P0'/AL2/16 | 0.047 | 0% | - | 10.0 | BLOD |
|  |  | 17 | B16/P0'/AL2/17 | 0.043 | 2% | - | 10.0 | BLOD |
|  |  | 18 | B16/P30'/AL1/18 | 0.044 | 3% | - | 10.0 | BLOD |
|  |  | 19 | B16/P30'/AL1/19 | 0.046 | 0% | - | 10.0 | BLOD |
|  |  | 20 | B16/P60'/AL2/20 | 0.047 | 0% | - | 10.0 | BLOD |
| GP5 | PP MIX. 150mg/Kg (from D23) | 22 | B16/P120'/AL2/22 | 0.045 | 2% | - | 10.0 | BLOD |
|  |  | 24 | B16/P240'/AL1/24 | 0.048 | 4% | - | 10.0 | BLOD |
|  |  | 25 | B16/P240'/AL2/25 | 0.046 | 0% | - | 10.0 | BLOD |
| GP6 | PP MIX. 200mg/Kg (from D0) | 26 | B16/P0'/AL2/26 | 0.046 | 5% | - | 10.0 | BLOD |
|  |  | 27 | **B16/P0'/AL2/27** | 0.048 | 4% | - | **14.3** | BLOD |
|  |  | 28 | **B16/P30'/AL2/28** | 0.046 | 2% | - | **12.5** | BLOD |
|  |  | 29 | **B16/P30'/AL2/29** | 0.045 | 2% | - | **14.3** | BLOD |
|  |  | 30 | B16/P60'/AL2/30 | 0.048 | 0% | - | 10.0 | BLOD |
| GP7 | PP MIX. 200mg/Kg (from D23) | 31 | B16/P60'/AL1/31 | 0.045 | 2% | - | 10.0 | BLOD |
|  |  | 32 | B16/P120'/AL1/32 | 0.048 | 0% | - | 10.0 | BLOD |
|  |  | 33 | **B16/P120'/AL2/33** | 0.047 | 0% | - | **11.1** | BLOD |
|  |  | 34 | **B16/P240'/AL2/34** | 0.046 | 0% | - | **11.1** | BLOD |
|  |  | 35 | B16/P240'/AL2/35 | 0.045 | 0% | - | 10.0 | BLOD |
| GP8 | CONTROL | 37 | **B16/P/AL1/37** | 0.046 | 2% | - | **12.5** | BLOD |
|  |  | 39 | B16/P/AL2/39 | 0.045 | 2% | - | 10.0 | BLOD |
|  |  | 40 | B16/P/AL2/40 | 0.047 | 0% | - | 10.0 | BLOD |

**Table S4. ELISA kit for *in vivo* analysis.** ELISA kits used to evaluate the anti-inflammatory effect of Pp1:2 *in vivo* and their performance characteristics.

| **ELISA kit** | **Catalog number** | **Lot number** | **Sensitivity** | **Detection range** |
| --- | --- | --- | --- | --- |
| IL-6  (Interleukin 6) ELISA KIT | Cat E-EL-M0044 | Lot zhtc2np0XV | 18.75pg/mL | 31.25-2000 pg/mL |
| IL-1b  (Interleukin 1 beta) ELISA KIT | Cat E-EL-M0037 | Lot. 7eyf3uv8L2 | 4.69pg/mL | 7.81-500 pg/mL |
| IL-17A   (Interleukin 17A) ELISA KIT | Cat E-EL-M0047 | Lot y4C8whe808 | 9.38pg/mL | 15.63-1000pg/mL |

**Figure S1.** Cellular viability assay of Pp1:2 on IL-1β-stimulated synoviocytes.

**Figure S2.** Metabolite analyses of pinobanksin, chrysin, galangin, pinocembrin and caffeic acid phenetyl ester in mouse serum samples.

**Figure S3.** Control of water and food consumption and body weight in mice.

**Figure S4. Effects of Pp1:2 treatment on collagen immunostaining.** Representative immunofluorescence images of collagen IV (red) and nuclei (DAPI, blue) in hind paw joint tissue across experimental groups. Bar graph showing mean fluorescence intensity (MFI) quantification (arbitrary units). One-way ANOVA with Bonferroni post hoc comparison was used for comparisons between groups. **p* < 0.05; ***p* < 0.01.
